# Tetra­yttrium difluoride disilicate orthosilicate, Y_4_F_2_[Si_2_O_7_][SiO_4_]

**DOI:** 10.1107/S1600536813026391

**Published:** 2013-09-28

**Authors:** Marion C. Schäfer, Ingo Hartenbach, Thomas Schleid

**Affiliations:** a Department of Chemistry and Biochemistry, University of Delaware, 304A Drake Hall, Newark, DE 19716, USA; b Institut für Anorganische Chemie, Universität Stuttgart, Pfaffenwaldring 55, 70569 Stuttgart, Germany

## Abstract

In the crystal structure of Y_4_F_2_[Si_2_O_7_][SiO_4_], three fundamental building blocks are present, *viz*. anionic disilicate and orthosilicate units ([Si_2_O_7_]^6−^ and [SiO_4_]^4−^) and cationic [F_2_Y_4_]^10+^ entities. The latter are built up by two [FY_3_]^8+^ triangles sharing a common edge. The four crystallographically independent Y^3+^ cations display coordination numbers of eight for one and of seven for the other three cations, provided by oxide and fluoride anions. The overall arrangement of the building blocks can be considered as layer-like parallel to the *ac* plane.

## Related literature

For isotypic Er_4_F_2_[Si_2_O_7_][SiO_4_], see: Müller-Bunz & Schleid (2001 ▶). For the minor by-product phase Y_3_F[Si_3_O_10_], see: Müller-Bunz & Schleid (1998 ▶). For the crystal structure of allanite (old name orthite), see: Rumanova & Nikoleva (1959 ▶).

## Experimental

### 

#### Crystal data

Y_4_F_2_[Si_2_O_7_][SiO_4_]
*M* *_r_* = 653.91
Triclinic, 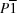

*a* = 6.4987 (5) Å
*b* = 6.6196 (5) Å
*c* = 13.2978 (9) Å
α = 87.418 (4)°
β = 85.702 (4)°
γ = 60.854 (3)°
*V* = 498.19 (6) Å^3^
*Z* = 2
Mo *K*α radiation
μ = 23.52 mm^−1^
*T* = 293 K
0.10 × 0.06 × 0.03 mm

#### Data collection

Nonius KappaCCD diffractometer
Absorption correction: numerical (*X-SHAPE*; Stoe & Cie, 1995 ▶) *T*_min_ = 0.104, *T*_max_ = 0.463
12473 measured reflections
2427 independent reflections
1475 reflections with *I* > 2σ(*I*)
*R*_int_ = 0.120

#### Refinement

*R*[*F* ^2^ > 2σ(*F* ^2^)] = 0.055
*wR*(*F* ^2^) = 0.107
*S* = 0.98
2427 reflections
182 parameters
Δρ_max_ = 1.56 e Å^−3^
Δρ_min_ = −1.49 e Å^−3^

### 

Data collection: *COLLECT* (Nonius, 1998 ▶); cell refinement: *SCALEPACK* (Otwinowski & Minor, 1997 ▶); data reduction: *SCALEPACK* and *DENZO* (Otwinowski & Minor, 1997 ▶); program(s) used to solve structure: *SHELXS97* (Sheldrick, 2008 ▶); program(s) used to refine structure: *SHELXL97* (Sheldrick, 2008 ▶); molecular graphics: *DIAMOND* (Brandenburg, 2006 ▶); software used to prepare material for publication: *SHELXL97*.

## Supplementary Material

Crystal structure: contains datablock(s) I, publication_text. DOI: 10.1107/S1600536813026391/wm2768sup1.cif

Structure factors: contains datablock(s) I. DOI: 10.1107/S1600536813026391/wm2768Isup2.hkl

Additional supplementary materials:  crystallographic information; 3D view; checkCIF report

## Figures and Tables

**Fig. 1.**
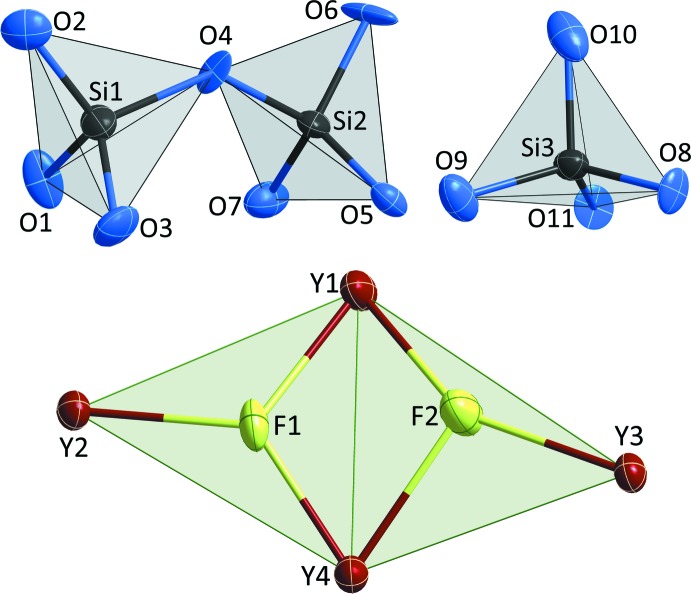
Disilicate ([Si2O7]6–: top left) and orthosilicate units ([SiO4]4–: top right) as well as butterfly-shaped cationic [F2Y4]10+ entities (bottom) in the crystal structure of Y4F2[Si2O7][SiO4]; displacement ellipsoids are drawn at the 80 % probability level.

**Fig. 2.**
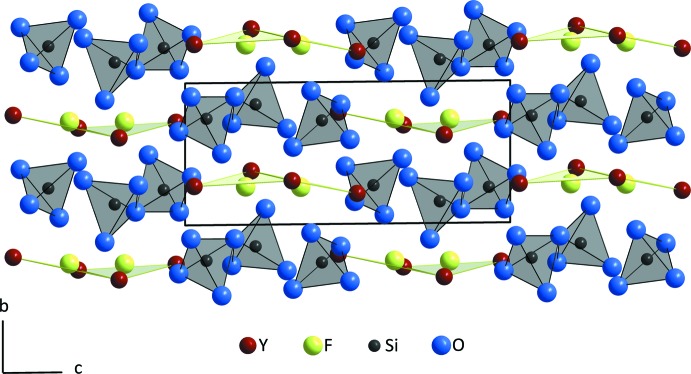
View at the crystal structure of Y4F2[Si2O7][SiO4] along [100], emphasizing the layer-like arrangement as line-up of the cationic and anionic building blocks.

## supplementary crystallographic information

### 1. Comment

Y_4_F_2_[Si_2_O_7_][SiO_4_] crystallizes isotypically with the already known erbium analogue Er_4_F_2_[Si_2_O_7_][SiO_4_] (Müller-Bunz & Schleid, 2001). The crystal structure comprises two different oxidosilicate anions, namely a pyroanionic bitetrahedral disilicate unit [Si_2_O_7_]^6–^ with eclipsed conformation (Fig. 1, *top left*) and an orthosilicate tetrahedron [SiO_4_]^4–^ (Fig. 1, *top right*), just like in the mineral allanite (old name orthite) (Rumanova & Nikoleva, 1959). Together with these two anionic building blocks, discrete cationic [F_2_Y_4_]^10+^ entities (Fig. 1, *bottom*) complete the crystal structure of Y_4_F_2_[Si_2_O_7_][SiO_4_]. For the formation of the latter, two almost planar [FY_3_]^8+^ triangles are fused together via one common edge, resulting in a butterfly-shaped [F_2_Y_4_]^10+^ unit comprising an angle between the two triangular planes of 161.65 (5)°. Two of the four crystallographically distinct Y^3+^ cations (Y2, Y3) display just one fluoride anion in their coordination sphere, while the other two (F1, F4) have contact with two F^–^ anions each. O^2–^ anions complete the coordination environments of the yttrium cations resulting in distorted bi- (Y1) or monocapped (Y2-4) trigonal prisms. The cationic [F_2_Y_4_]^10+^ as well as the anionic [Si_2_O_7_]^6–^ and [SiO_4_]^4–^ building blocks are arranged layer-like parallel to the *ac* plane in the crystal structure of the title compound (Fig. 2).

### 2. Experimental

Colourless lath-shaped single crystals of Y_4_F_2_[Si_2_O_7_][SiO_4_] were obtained by the reaction of yttrium sesquioxide (Y_2_O_3_), yttrium trifluoride (YF_3_), and silicon dioxide (SiO_2_) in the molar ratio 2:5:3 and an excess of cesium chloride (CsCl) as flux in evacuated silica ampoules within nine days at 973 K and a cooling rate of 10 Kh^-1^. Due to the stability of the product against air and moisture, the excess flux can the removed by washing with water. Besides the title compound, single crystals of thalenite-type Y_3_F[Si_3_O_10_] (Müller-Bunz & Schleid, 1998) were also found in the product mixture as minor by-product.

### 3. Refinement

The highest and lowest electron densities are found 1.29 Å from atom F2 and and 1.28 Å from atom O8, respectively.

### Crystal data

**Table:**

Y_4_F_2_[Si_2_O_7_][SiO_4_]	*Z* = 2
*M**_r_* = 653.91	*F*(000) = 608
Triclinic, *P*1	*D*_x_ = 4.359 Mg m^−^^3^
Hall symbol: -P 1	Mo *K*α radiation, λ = 0.71073 Å
*a* = 6.4987 (5) Å	Cell parameters from 5136 reflections
*b* = 6.6196 (5) Å	θ = 0.4–28.3°
*c* = 13.2978 (9) Å	µ = 23.52 mm^−^^1^
α = 87.418 (4)°	*T* = 293 K
β = 85.702 (4)°	Lath-shaped, colourless
γ = 60.854 (3)°	0.10 × 0.06 × 0.03 mm
*V* = 498.19 (6) Å^3^	

### Data collection

**Table:**

Nonius KappaCCD diffractometer	2427 independent reflections
Radiation source: fine-focus sealed tube	1475 reflections with *I* > 2σ(*I*)
Graphite monochromator	*R*_int_ = 0.120
ω and φ mscans	θ_max_ = 28.2°, θ_min_ = 1.5°
Absorption correction: numerical (*X-SHAPE*; Stoe & Cie, 1995)	*h* = −8→8
*T*_min_ = 0.104, *T*_max_ = 0.463	*k* = −8→8
12473 measured reflections	*l* = −17→17

### Refinement

**Table:**

Refinement on *F*^2^	Primary atom site location: structure-invariant direct methods
Least-squares matrix: full	Secondary atom site location: difference Fourier map
*R*[*F*^2^ > 2σ(*F*^2^)] = 0.055	*w* = 1/[σ^2^(*F*_o_^2^) + (0.0233*P*)^2^] where *P* = (*F*_o_^2^ + 2*F*_c_^2^)/3
*wR*(*F*^2^) = 0.107	(Δ/σ)_max_ < 0.001
*S* = 0.98	Δρ_max_ = 1.56 e Å^−^^3^
2427 reflections	Δρ_min_ = −1.49 e Å^−^^3^
182 parameters	Extinction correction: *SHELXL97* (Sheldrick, 2008), Fc^*^=kFc[1+0.001xFc^2^λ^3^/sin(2θ)]^-1/4^
0 restraints	Extinction coefficient: 0.0029 (6)

### Special details

**Table:**

Geometry. All esds (except the esd in the dihedral angle between two l.s. planes) are estimated using the full covariance matrix. The cell esds are taken into account individually in the estimation of esds in distances, angles and torsion angles; correlations between esds in cell parameters are only used when they are defined by crystal symmetry. An approximate (isotropic) treatment of cell esds is used for estimating esds involving l.s. planes.
Refinement. Refinement of F^2^ against ALL reflections. The weighted R-factor wR and goodness of fit S are based on F^2^, conventional R-factors R are based on F, with F set to zero for negative F^2^. The threshold expression of F^2^ > 2sigma(F^2^) is used only for calculating R-factors(gt) etc. and is not relevant to the choice of reflections for refinement. R-factors based on F^2^ are statistically about twice as large as those based on F, and R- factors based on ALL data will be even larger.

### Fractional atomic coordinates and isotropic or equivalent isotropic displacement parameters (Å^2^)

**Table:**

	*x*	*y*	*z*	*U*_iso_*/*U*_eq_	
Y1	0.32290 (19)	0.37903 (19)	0.20317 (8)	0.0102 (3)	
Y2	0.94009 (19)	0.27320 (19)	0.02756 (8)	0.0083 (3)	
Y3	0.21943 (19)	0.21311 (19)	0.52890 (8)	0.0083 (3)	
Y4	0.82014 (19)	0.30705 (19)	0.32901 (8)	0.0084 (3)	
F1	0.0366 (11)	0.2679 (11)	0.1862 (5)	0.0118 (14)	
F2	0.2019 (11)	0.2616 (11)	0.3554 (5)	0.0143 (15)	
Si1	0.4925 (5)	0.2508 (5)	0.9343 (2)	0.0098 (7)	
Si2	0.2335 (5)	0.1526 (5)	0.7833 (2)	0.0084 (7)	
Si3	0.2675 (5)	0.7298 (5)	0.4207 (2)	0.0078 (7)	
O1	0.2654 (13)	0.3396 (14)	0.0197 (6)	0.0120 (17)	
O2	0.2523 (13)	0.9039 (13)	0.0181 (6)	0.0117 (17)	
O3	0.5550 (14)	0.5023 (14)	0.1127 (6)	0.0145 (18)	
O4	0.4646 (12)	0.0987 (13)	0.8473 (5)	0.0086 (16)	
O5	0.2042 (13)	0.3248 (13)	0.6867 (5)	0.0081 (16)	
O6	0.6842 (13)	0.1117 (13)	0.2472 (6)	0.0122 (17)	
O7	0.0069 (13)	0.7152 (13)	0.1437 (6)	0.0116 (17)	
O8	0.4064 (13)	0.8414 (13)	0.4786 (6)	0.0092 (16)	
O9	0.4233 (13)	0.5444 (14)	0.3321 (6)	0.0132 (18)	
O10	0.8272 (13)	0.4057 (14)	0.4931 (6)	0.0135 (18)	
O11	0.0157 (13)	0.9376 (13)	0.3873 (6)	0.0089 (17)	

### Atomic displacement parameters (Å^2^)

**Table:**

	*U*^11^	*U*^22^	*U*^33^	*U*^12^	*U*^13^	*U*^23^
Y1	0.0090 (5)	0.0126 (6)	0.0088 (6)	−0.0050 (4)	−0.0027 (4)	0.0019 (4)
Y2	0.0088 (5)	0.0079 (6)	0.0077 (6)	−0.0035 (4)	−0.0013 (4)	−0.0008 (4)
Y3	0.0082 (5)	0.0081 (6)	0.0078 (6)	−0.0032 (4)	−0.0023 (4)	−0.0001 (4)
Y4	0.0079 (5)	0.0088 (6)	0.0076 (6)	−0.0034 (4)	−0.0011 (4)	−0.0008 (4)
F1	0.014 (3)	0.013 (4)	0.009 (3)	−0.005 (3)	−0.005 (3)	−0.001 (3)
F2	0.013 (3)	0.019 (4)	0.012 (4)	−0.009 (3)	−0.004 (3)	0.003 (3)
Si1	0.0073 (15)	0.0095 (16)	0.0107 (16)	−0.0022 (13)	−0.0022 (12)	−0.0016 (12)
Si2	0.0083 (15)	0.0058 (16)	0.0077 (16)	−0.0005 (13)	−0.0002 (12)	−0.0019 (12)
Si3	0.0079 (15)	0.0083 (16)	0.0077 (16)	−0.0044 (13)	0.0006 (12)	0.0002 (12)
O1	0.016 (4)	0.019 (4)	0.009 (4)	−0.009 (3)	0.005 (3)	−0.003 (3)
O2	0.011 (4)	0.010 (4)	0.016 (4)	−0.006 (3)	0.001 (3)	0.001 (3)
O3	0.017 (4)	0.012 (4)	0.010 (4)	−0.004 (3)	−0.003 (3)	0.006 (3)
O4	0.009 (4)	0.013 (4)	0.011 (4)	−0.006 (3)	−0.005 (3)	0.003 (3)
O5	0.010 (4)	0.009 (4)	0.009 (4)	−0.005 (3)	0.002 (3)	−0.002 (3)
O6	0.009 (4)	0.009 (4)	0.019 (4)	−0.001 (3)	−0.001 (3)	−0.004 (3)
O7	0.011 (4)	0.010 (4)	0.009 (4)	−0.002 (3)	0.002 (3)	0.002 (3)
O8	0.009 (4)	0.010 (4)	0.012 (4)	−0.006 (3)	−0.004 (3)	−0.002 (3)
O9	0.014 (4)	0.019 (4)	0.009 (4)	−0.008 (3)	0.000 (3)	−0.004 (3)
O10	0.012 (4)	0.012 (4)	0.009 (4)	0.000 (3)	−0.003 (3)	−0.001 (3)
O11	0.009 (4)	0.013 (4)	0.010 (4)	−0.005 (3)	−0.006 (3)	0.001 (3)

### Geometric parameters (Å, º)

**Table:**

Y1—O6	2.248 (7)	Y3—Si3^ii^	3.024 (3)
Y1—O3	2.286 (8)	Y3—Si2	3.392 (3)
Y1—O7	2.330 (7)	Y3—Y3^i^	3.406 (2)
Y1—F1	2.337 (6)	Y3—Si3^x^	3.431 (3)
Y1—F2	2.353 (6)	Y3—Y3^ii^	3.559 (2)
Y1—O9	2.366 (7)	Y4—F1^iv^	2.223 (6)
Y1—O1	2.540 (8)	Y4—O6	2.240 (8)
Y1—O4^i^	2.856 (8)	Y4—O11^v^	2.269 (8)
Y1—Si2^i^	3.296 (3)	Y4—O9	2.272 (8)
Y1—Si2^ii^	3.428 (3)	Y4—O10	2.316 (8)
Y1—Y4	3.5649 (15)	Y4—O5^vi^	2.364 (7)
Y1—Y2^iii^	3.7351 (15)	Y4—F2^iv^	2.400 (6)
Y2—O2^iii^	2.216 (8)	Y4—Si3^vi^	3.352 (3)
Y2—F1^iv^	2.239 (6)	Y4—Y3^vi^	3.6603 (16)
Y2—O7^iii^	2.281 (8)	Y4—Y3^iv^	3.6740 (14)
Y2—O2^v^	2.295 (7)	Y4—Y1^iv^	3.7852 (15)
Y2—O1^iii^	2.322 (8)	F1—Y4^xi^	2.223 (6)
Y2—O1^iv^	2.354 (7)	F1—Y2^xi^	2.239 (6)
Y2—O3	2.424 (8)	F2—Y4^xi^	2.400 (6)
Y2—Si1^vi^	3.064 (3)	Si1—O3^vi^	1.613 (8)
Y2—Si1^vii^	3.316 (3)	Si1—O2^vi^	1.626 (8)
Y2—Y2^viii^	3.411 (2)	Si1—O4	1.644 (8)
Y2—Y2^ix^	3.486 (2)	Si1—O1^xii^	1.666 (8)
Y2—Y1^iii^	3.7351 (15)	Si2—O6^i^	1.622 (8)
Y3—O5	2.236 (7)	Si2—O7^ii^	1.633 (8)
Y3—O8^x^	2.257 (8)	Si2—O5	1.637 (8)
Y3—O8^vi^	2.273 (7)	Si2—O4	1.657 (7)
Y3—O10^xi^	2.305 (7)	Si3—O11	1.621 (7)
Y3—F2	2.319 (6)	Si3—O9	1.632 (8)
Y3—O11^ii^	2.388 (8)	Si3—O8	1.660 (7)
Y3—O10^vi^	2.398 (8)	Si3—O10^vi^	1.684 (8)
			
O6—Y1—O3	78.8 (3)	O6—Y4—O10	138.3 (3)
O6—Y1—O7	163.3 (3)	O11^v^—Y4—O10	84.6 (3)
O3—Y1—O7	85.3 (3)	O9—Y4—O10	90.6 (3)
O6—Y1—F1	119.2 (2)	F1^iv^—Y4—O5^vi^	78.7 (2)
O3—Y1—F1	142.5 (2)	O6—Y4—O5^vi^	136.0 (3)
O7—Y1—F1	76.9 (2)	O11^v^—Y4—O5^vi^	148.0 (3)
O6—Y1—F2	83.0 (3)	O9—Y4—O5^vi^	78.4 (3)
O3—Y1—F2	151.4 (2)	O10—Y4—O5^vi^	75.9 (3)
O7—Y1—F2	109.4 (2)	F1^iv^—Y4—F2^iv^	67.0 (2)
F1—Y1—F2	66.0 (2)	O6—Y4—F2^iv^	135.7 (3)
O6—Y1—O9	73.5 (3)	O11^v^—Y4—F2^iv^	77.8 (2)
O3—Y1—O9	79.2 (3)	O9—Y4—F2^iv^	147.8 (3)
O7—Y1—O9	98.5 (3)	O10—Y4—F2^iv^	70.5 (2)
F1—Y1—O9	135.6 (2)	O5^vi^—Y4—F2^iv^	71.9 (2)
F2—Y1—O9	74.6 (2)	Y4^xi^—F1—Y2^xi^	128.6 (3)
O6—Y1—O1	111.4 (3)	Y4^xi^—F1—Y1	112.2 (2)
O3—Y1—O1	75.0 (3)	Y2^xi^—F1—Y1	114.7 (3)
O7—Y1—O1	68.9 (3)	Y3—F2—Y1	149.1 (3)
F1—Y1—O1	67.9 (2)	Y3—F2—Y4^xi^	102.2 (2)
F2—Y1—O1	132.7 (2)	Y1—F2—Y4^xi^	105.6 (2)
O9—Y1—O1	152.0 (3)	O3^vi^—Si1—O2^vi^	115.4 (4)
O6—Y1—O4^i^	56.4 (2)	O3^vi^—Si1—O4	109.4 (4)
O3—Y1—O4^i^	103.4 (3)	O2^vi^—Si1—O4	108.6 (4)
O7—Y1—O4^i^	133.9 (2)	O3^vi^—Si1—O1^xii^	99.6 (4)
F1—Y1—O4^i^	68.9 (2)	O2^vi^—Si1—O1^xii^	113.6 (4)
F2—Y1—O4^i^	84.1 (2)	O4—Si1—O1^xii^	110.0 (4)
O9—Y1—O4^i^	127.5 (2)	O6^i^—Si2—O7^ii^	117.1 (4)
O1—Y1—O4^i^	70.1 (2)	O6^i^—Si2—O5	114.0 (4)
O2^iii^—Y2—F1^iv^	122.9 (3)	O7^ii^—Si2—O5	106.6 (4)
O2^iii^—Y2—O7^iii^	79.4 (3)	O6^i^—Si2—O4	97.8 (4)
F1^iv^—Y2—O7^iii^	154.6 (3)	O7^ii^—Si2—O4	109.3 (4)
O2^iii^—Y2—O2^v^	81.8 (3)	O5—Si2—O4	111.9 (4)
F1^iv^—Y2—O2^v^	85.6 (2)	O11—Si3—O9	114.6 (4)
O7^iii^—Y2—O2^v^	86.0 (3)	O11—Si3—O8	109.0 (4)
O2^iii^—Y2—O1^iii^	108.9 (3)	O9—Si3—O8	115.8 (4)
F1^iv^—Y2—O1^iii^	106.1 (2)	O11—Si3—O10^vi^	99.8 (4)
O7^iii^—Y2—O1^iii^	73.6 (3)	O9—Si3—O10^vi^	107.4 (4)
O2^v^—Y2—O1^iii^	154.3 (3)	O8—Si3—O10^vi^	108.9 (4)
O2^iii^—Y2—O1^iv^	153.3 (3)	Si1^vii^—O1—Y2^iii^	99.1 (4)
F1^iv^—Y2—O1^iv^	72.8 (2)	Si1^vii^—O1—Y2^xi^	129.2 (4)
O7^iii^—Y2—O1^iv^	82.0 (3)	Y2^iii^—O1—Y2^xi^	96.4 (3)
O2^v^—Y2—O1^iv^	78.1 (3)	Si1^vii^—O1—Y1	120.2 (4)
O1^iii^—Y2—O1^iv^	83.6 (3)	Y2^iii^—O1—Y1	100.3 (3)
O2^iii^—Y2—O3	78.4 (3)	Y2^xi^—O1—Y1	103.8 (3)
F1^iv^—Y2—O3	78.6 (2)	Si1^vi^—O2—Y2^iii^	118.5 (4)
O7^iii^—Y2—O3	121.1 (3)	Si1^vi^—O2—Y2^xiii^	139.1 (5)
O2^v^—Y2—O3	142.0 (3)	Y2^iii^—O2—Y2^xiii^	98.2 (3)
O1^iii^—Y2—O3	63.6 (3)	Si1^vi^—O3—Y1	133.6 (5)
O1^iv^—Y2—O3	127.9 (3)	Si1^vi^—O3—Y2	96.7 (4)
O5—Y3—O8^x^	124.4 (3)	Y1—O3—Y2	128.7 (3)
O5—Y3—O8^vi^	84.1 (3)	Si1—O4—Si2	130.4 (5)
O8^x^—Y3—O8^vi^	82.5 (3)	Si1—O4—Y1^i^	136.4 (4)
O5—Y3—O10^xi^	102.1 (3)	Si2—O4—Y1^i^	89.8 (3)
O8^x^—Y3—O10^xi^	112.7 (3)	Si2—O5—Y3	121.5 (4)
O8^vi^—Y3—O10^xi^	154.3 (3)	Si2—O5—Y4^vi^	132.8 (4)
O5—Y3—F2	155.0 (2)	Y3—O5—Y4^vi^	105.4 (3)
O8^x^—Y3—F2	79.2 (3)	Si2^i^—O6—Y4	138.9 (4)
O8^vi^—Y3—F2	91.6 (2)	Si2^i^—O6—Y1	115.8 (4)
O10^xi^—Y3—F2	72.2 (2)	Y4—O6—Y1	105.2 (3)
O5—Y3—O11^ii^	79.8 (3)	Si2^ii^—O7—Y2^iii^	129.9 (4)
O8^x^—Y3—O11^ii^	77.4 (3)	Si2^ii^—O7—Y1	118.8 (4)
O8^vi^—Y3—O11^ii^	140.4 (3)	Y2^iii^—O7—Y1	108.2 (3)
O10^xi^—Y3—O11^ii^	65.2 (3)	Si3—O8—Y3^xiv^	121.6 (4)
F2—Y3—O11^ii^	117.1 (2)	Si3—O8—Y3^vi^	135.4 (4)
O5—Y3—O10^vi^	76.7 (3)	Y3^xiv^—O8—Y3^vi^	97.5 (3)
O8^x^—Y3—O10^vi^	147.8 (3)	Si3—O9—Y4	125.0 (4)
O8^vi^—Y3—O10^vi^	75.5 (3)	Si3—O9—Y1	133.0 (4)
O10^xi^—Y3—O10^vi^	81.6 (3)	Y4—O9—Y1	100.4 (3)
F2—Y3—O10^vi^	78.4 (3)	Si3^vi^—O10—Y3^iv^	97.4 (3)
O11^ii^—Y3—O10^vi^	133.8 (3)	Si3^vi^—O10—Y4	112.9 (4)
F1^iv^—Y4—O6	84.0 (3)	Y3^iv^—O10—Y4	105.3 (3)
F1^iv^—Y4—O11^v^	99.0 (3)	Si3^vi^—O10—Y3^vi^	136.1 (4)
O6—Y4—O11^v^	74.4 (3)	Y3^iv^—O10—Y3^vi^	98.4 (3)
F1^iv^—Y4—O9	119.3 (2)	Y4—O10—Y3^vi^	101.9 (3)
O6—Y4—O9	75.5 (3)	Si3—O11—Y4^xiii^	145.4 (4)
O11^v^—Y4—O9	127.5 (3)	Si3—O11—Y3^ii^	96.1 (4)
F1^iv^—Y4—O10	135.4 (2)	Y4^xiii^—O11—Y3^ii^	116.6 (3)

Symmetry codes: (i) −*x*+1, −*y*, −*z*+1; (ii) −*x*, −*y*+1, −*z*+1; (iii) −*x*+1, −*y*+1, −*z*; (iv) *x*+1, *y*, *z*; (v) *x*+1, *y*−1, *z*; (vi) −*x*+1, −*y*+1, −*z*+1; (vii) *x*, *y*, *z*−1; (viii) −*x*+2, −*y*, −*z*; (ix) −*x*+2, −*y*+1, −*z*; (x) *x*, *y*−1, *z*; (xi) *x*−1, *y*, *z*; (xii) *x*, *y*, *z*+1; (xiii) *x*−1, *y*+1, *z*; (xiv) *x*, *y*+1, *z*.
